# Accuracy of the recording of pneumonia events in English electronic healthcare record data in patients with chronic obstructive pulmonary disease

**DOI:** 10.1186/s41479-024-00130-2

**Published:** 2024-05-05

**Authors:** Alexander J. Adamson, Constantinos Kallis, Ian Douglas, Jennifer K. Quint

**Affiliations:** 1 Imperial College London, London, UK; 2 London School of Hygiene and Tropical Medicine, London, UK

## Abstract

**Background:**

In primary care, identifying pneumonia events in people with chronic obstructive pulmonary disease (COPD) may be challenging due to similarities in symptoms with COPD exacerbations and lack of diagnostic testing. This study explored the accuracy of pneumonia diagnosis coded in primary care by comparing diagnosis in primary care with diagnosis in hospital.

**Methods:**

A study population of people with COPD in England was created using the Clinical Practice Research Datalink Aurum database linked with Hospital Episode Statistics inpatient data. Pneumonia codes only, and pneumonia code with associated clinical and/or treatment codes (chest x-ray, symptoms, antibiotics, sputum and blood culture) were used to determine pneumonia events in primary care. Events that were followed by hospitalisation within 7 days were used to estimate the positive predictive value (PPV) of pneumonia coding in primary care, using primary diagnosis of pneumonia in secondary care as the gold standard. The PPV of primary care recording of hospitalised pneumonia was also calculated.

**Results:**

Two hundred seventy-four thousand one hundred fifty-six COPD patients were eligible for inclusion, of whom 7,560 had an eligible pneumonia event in primary care diagnosed between 2015–2019 which was not ‘hospital-acquired’ and was diagnosed and entered on the same day. Of the 2,094 events which were followed by hospitalisation within 7 days, 1,208 had a primary diagnosis of pneumonia in hospital, representing a PPV of pneumonia coding in primary care of 57.7% (95% CI 55.6%-59.8%). Another 284 (13.6%) were diagnosed as a COPD exacerbation and 114 (5.4%) were diagnosed as another respiratory disease. Use of additional pneumonia clinical and treatment codes had a modest effect on the PPV but substantially lowered the number of events. Of the 33,603 eligible pneumonia events identified in secondary care, only 11,445 were recorded in primary care within 42 days, representing a sensitivity of 34.1% (95% CI 33.6%-34.6%).

**Conclusions:**

Use of primary care pneumonia codes and associated clinical and treatment codes to determine pneumonia is not recommended due to significant levels of misdiagnosis and many hospitalised events failing to be recorded in primary care.

**Supplementary Information:**

The online version contains supplementary material available at 10.1186/s41479-024-00130-2.

## Introduction

Chronic obstructive pulmonary disease (COPD) affects around 3 million people in the UK and is responsible for 140,000 admissions and 30,000 deaths per year [1]. The most common cause is smoking, and patients exhibit airflow obstruction that is not fully reversible [2]. The disease is progressive, with declining lung function and a worsening of symptoms over time. COPD patients may experience acute exacerbations which manifest as a sudden worsening of symptoms. 50–70% of exacerbations are thought to be caused by infections [3]. Exacerbations of COPD are an important cause of hospital admission and readmission which may have considerable impact on patients’ quality of life and activities of daily living.

Pneumonia is another common lung disease, affecting around 0.5–1% of British adults each year [4]. Pneumonia is an inflammation of the alveoli in one or both of the lungs that is usually caused by infection by a virus or bacteria [5]. Symptoms range from moderate to severe, with moderate symptoms managed at home with antibiotics but more severe symptoms requiring hospital admission. Pneumonia causes around 200,000 hospital admissions and 29,000 deaths per year, making it the 6^th^ largest cause of mortality in the UK [1].

The risk of contracting pneumonia is higher among individuals with COPD [6], and pneumonia is an important cause of hospital admission and readmission in this population. Diagnosing community-acquired pneumonia (CAP) in patients with COPD poses a challenge given the overlap of symptoms with an exacerbation. Whilst technically pneumonia is a sub-type of lower respiratory tract infection (LRTI) [7], in practice pneumonia is coded and treated differently and warrants its own separate diagnosis. Definitive diagnosis of pneumonia requires a chest X-ray, which may be more difficult to access from primary care settings [8]. Due to the overlapping clinical presentations and the British Thoracic Society (BTS) guidelines advising against rigorous differentiation between LRTIs and pneumonia [8] for the purpose of labelling disease, there exists a significant potential for misdiagnosis.

Routinely collected electronic health and administrative data of patients is a valuable tool for health and epidemiological research. The validity and generalisability of any research findings using patients’ electronic health records (EHR) depends on accurate diagnosis of disease outcomes.

Validation of various respiratory disease outcomes (e.g., COPD exacerbations) have been carried out in other studies [9]. However, there is a paucity of data around accurate determination of pneumonia events in EHR in COPD patients, a population in which it can be difficult for clinicians to differentiate pneumonia from an exacerbation. Furthermore, there has been a recent focus on the use of inhaled corticosteroids (ICS) and its association with pneumonia in COPD patients [10], adding to the importance of accurate diagnosis in this population in an epidemiological setting. Therefore, our main objective was to develop algorithms that would help to accurately identify pneumonia events in COPD patients in EHR. Pneumonia events recorded in Hospital Episode Statistics (HES) were used as the gold standard, as chest x-ray is recommended and available for all patients admitted to hospital with suspected pneumonia [8]. Initially, we tested algorithms that combined various clinical features and chest radiography to understand the best method of finding pneumonia events among COPD patients in primary care. Subsequently, we identified how well pneumonia diagnosed in secondary care was recorded in primary care.

## Methods

### Data sources

This study used routinely collected primary care data from GP practices using EMISWeb software, data which are curated by the UK’s Clinical Practice Research Datalink (CPRD) service and made available to researchers as the CPRD Aurum database. As of May 2021, CPRD Aurum included longitudinal health data for 13,351,330 current acceptable patients, representing 20% of the UK population [11]. Aurum data have been shown to be nationally representative, including with respect to age and sex [12]. Data in CPRD Aurum contains information on patient demographics, clinical diagnoses, consultations, primary care prescription medications, laboratory tests, and specialist referrals. Linked socioeconomic data from the Index of Multiple Deprivation (IMD), and secondary care data covering accident and emergency (A&E) attendances and admissions to hospital from Hospital Episode Statistics (HES) were provided for this study by CPRD. Approximately 75% of CPRD practices in England are eligible for linkage [12].

### Study population

COPD patients were eligible for inclusion if they met the following criteria: 1) had a diagnosis of COPD using validated codes [13]; 2) were aged 35 or older at COPD diagnosis 3) were registered at a GP practice between 1^st^ January 2015–31^st^ December 2019; 4) passed basic internal data consistency checks implemented at a practice and patient level by CPRD to ensure data is of suitable research quality [12]; and 5) were eligible for linkage to Hospital Episode Statistics data. Patients were eligible for linkage if they were based at practices in England that had not opted out of data linkage and had not opted out at a patient level. Pneumonia events were determined for eligible patients from a time period which started at the latest date of the following: 1) 1^st^ January 2015; 2) diagnosed with COPD for at least 1 year; or 3) registration date at practice. The time period for identifying pneumonia events ended at the earliest of the following: 1) 31^st^ December 2019; 2) death; 3) transfer out from the practice; or 4) last collection date from the practice.

### Outcome

The main outcome of interest was a pneumonia event. This was defined separately in HES (secondary care) and in CPRD Aurum (primary care). In secondary care, the primary code of the last episode was used to determine the primary reason for admission. The following international disease classification (ICD) ICD10 codes were used to define pneumonia admission: J12 (Viral pneumonia), J13 (Pneumonia due to *S. Pneumoniae*), J14 (Pneumonia due to *H. Influenzae*), J15 (Bacterial pneumonia not elsewhere classified), J16 (Pneumonia due to other infectious organism), J17 (Pneumonia in diseases classified elsewhere), J18 (Pneumonia: organism unspecified) [14]. Based on previous validation studies [15], we anticipated that the HES diagnosis would be accurate due to recommended use of chest X-ray to obtain a definitive diagnosis [8], and we used this as the gold standard.

To determine pneumonia diagnosis in primary care, a pneumonia codelist was developed using the search term ‘pneumonia’ to find all terms relating to pneumonia in the EMISWeb software. This codelist was then checked by a respiratory physician to remove irrelevant codes e.g. ambiguous codes such as ‘pneumonia or influenza nos’ were removed. There was also no overlap between the codes used in the validated COPD exacerbation codelist and the primary care pneumonia codelist. The codelist is provided in the Supplementary material and is available at https://github.com/NHLI-Respiratory-Epi/Pneumonia-Accuracy-EHR. For the first part of the study, in which the quality of pneumonia coding in primary care was validated using pneumonia coding in secondary care, pneumonia events were restricted to those on which the observation date and data entry date were the same to ensure prospective rather than retrospective coding to minimise the likelihood of secondary care events then being recorded in primary care. Furthermore, we explored the coding of pneumonia events in 19 pre-defined algorithms (Table 2). The following clinical features were used to define the study population algorithms; symptoms (at least two of the following symptoms: new cough, sputum, breathlessness, fever, lethargy, tachycardia), referrals for chest x-ray, antibiotics use, sputum sample and blood culture. The components of the predefined algorithms occurred within a 7-day window. The 7-day window of events was chosen because symptoms and other clinical features manifest between 3 to 7 days after infection. When assessing the quality of recording of hospital pneumonia events in primary care, we used a 42-day window to determine recording in primary care using the developed pneumonia codelist, with and without a same-day respiratory or generic hospitalisation code.

### Patient characteristics

Eligible patients had the following variables included: age at pneumonia diagnosis, sex, smoking status, IMD quintile, Body Mass Index (BMI) (derived by calculating patients’ weight in kilograms divided by height in meters squared and categorized as Underweight (Below 18.5), Normal (18.5–24.9), Overweight (25.0–29.9), and Obese (30.0 and greater) using WHO classifications for categories of BMI), blood pressure, diagnosis of hypertension, GOLD status (derived by calculating FEV1%-predicted and classifying into the four GOLD stages (stage 1: FEV1%-predicted > = 80%; stage 2 FEV1%-predicted 50–79%; stage 3 FEV1%-predicted 30–49%; stage 4 FEV1%-predicted < 30%), Charlson comorbidity index (CCI) diseases and counts, asthma diagnosis, anxiety diagnosis, depression diagnosis, oral corticosteroid use in the preceding 5 years, and COPD inhaler use in preceding 5 years (long-acting muscarinic antagonist (LAMA), long-acting beta agonist (LABA), inhaled corticosteroid (ICS), short-acting muscarinic antagonist (SAMA), short-acting beta agonist (SABA), LAMA-LABA dual therapy, ICS-LABA dual therapy, LAMA-LABA-ICS triple therapy). For patients who were admitted to hospital, the length of stay was also calculated. We used clinical codes as recorded in primary care to describe patients’ characteristics and clinical features, and product codes to describe patients’ prescriptions.

### Data analysis

To assess the quality of coding of pneumonia events identified in primary care, we restricted pneumonia-coded events in primary care to just those that resulted in hospitalisation within 7 days, and calculated the PPV of the various algorithms using diagnosis in hospital as the gold standard. The restriction to only hospitalised events was applied because only pneumonia events seen in primary care that result in hospitalisation can be compared with the gold standard of secondary care coding. Sensitivity analyses were performed whereby the gold standard HES diagnosis was defined as having a pneumonia code in any position in the last episode rather than the first position, and an additional analysis was performed whereby pneumonia events were restricted to just those that occur on the same day as hospital admission.

To assess the quality of coding of pneumonia hospitalisation in primary care, we determined pneumonia diagnoses in HES, and calculated sensitivity by looking forward to identify pneumonia records in primary care within 42 days of admission. Hospitalised pneumonia code in primary care was defined firstly as pneumonia code only, and secondly as pneumonia code with associated general or respiratory hospital admission code on the same day.

To estimate the diagnostic accuracy of our algorithms, we implemented exact binomial confidence intervals for sensitivity and PPV. For both sections, we estimated the frequency of the individual pneumonia codes used and an individual codes’ association with pneumonia in secondary care. Secondary care diagnoses were descriptively presented when secondary care diagnosis contradicted a diagnosis of pneumonia in primary care.

## Results

**Fig. 1:**
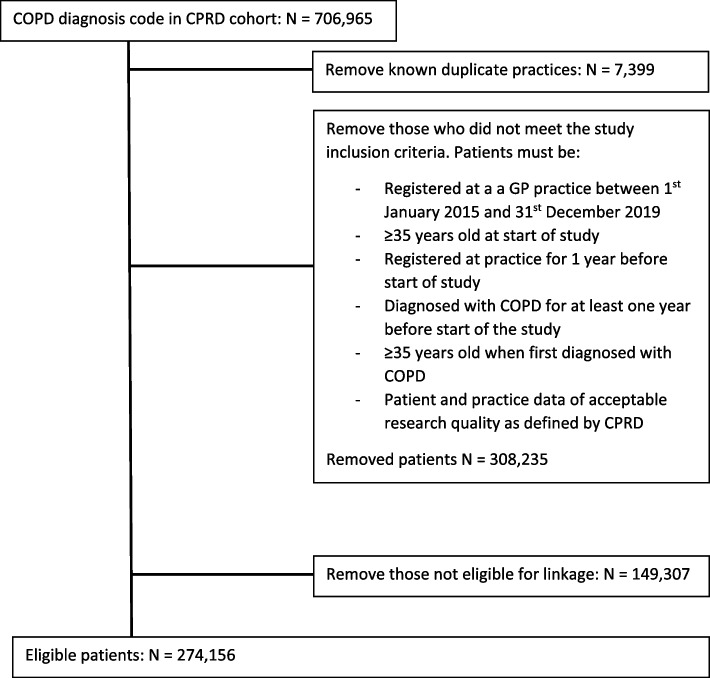
Flow chart displaying the route to eligibility for inclusion in the study

**Fig. 2:**
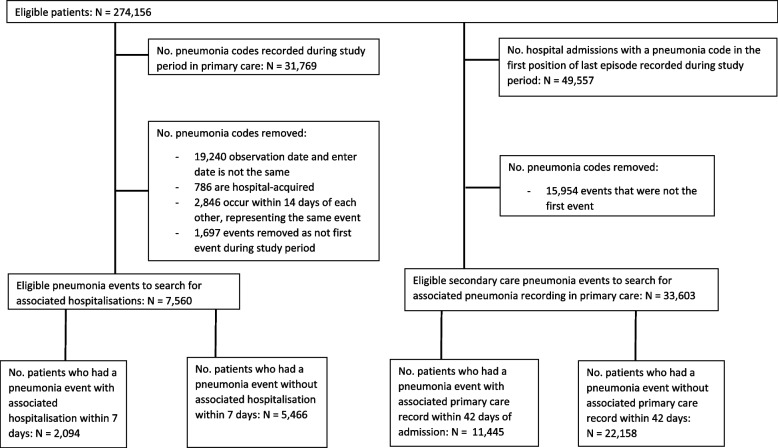
Flow chart demonstrating how eligible patients arrived in the group selected by primary care codes and secondary care codes

Out of the 706,965 patients with COPD in primary care, 274,156 patients remained eligible for inclusion in the study after applying the inclusion and exclusion criteria (Fig. 1). Of these eligible patients, 7,560 pneumonia events in primary care were eligible for inclusion in the study assessing accuracy of coding incident pneumonia cases in primary care, of which 2,094 patients were admitted to hospital (Fig. 2). When assessing the accuracy of recording hospitalised pneumonia in primary care, 33,603 secondary care pneumonia events were available for inclusion (Fig. 2).

The characteristics of patients who had an eligible pneumonia event in primary care are displayed in Table 1. Those who were admitted to hospital tended to be older, with greater numbers of comorbidities. Table 2 shows the PPV of each pneumonia algorithm on pneumonia diagnosis in hospital. More detailed algorithms tended to increase the PPV for pneumonia, but typically resulted in far fewer events identified overall, suggesting a lowered sensitivity. Pneumonia code, pneumonia code with chest X-ray referral, pneumonia code with any antibiotics prescription, and pneumonia code with an antibiotic prescription lasting 5–14 days were the only algorithms that resulted in > 100 hospital admissions overall, with PPVs ranging from 47.5 (95% CI 42.0–53.1) for those with a pneumonia code and antibiotic prescription lasting 5–14 days to 60.2 (95% CI 54.9–65.2) for those with a pneumonia code and referral for chest X-ray. Use of pneumonia code alone identified the most pneumonia events in hospital (1,208), with a PPV of 57.7 (95% CI 55.6–59.8). Of those with a pneumonia code in primary care who were admitted to secondary care with a primary diagnosis other than pneumonia, 284 (32.0%) had a primary diagnosis of COPD, 114 (12.9%) had a primary diagnosis of a respiratory disease other than COPD or pneumonia, and 109 (12.3%) had a primary diagnosis of a circulatory disease. The breakdown of primary care pneumonia codes that did and did not result in a primary diagnosis of pneumonia in secondary care can be found in Supplementary Fig. 1. Whilst there was no significant difference in the length of stay between those who received a primary diagnosis of pneumonia in hospital and those who received a primary diagnosis other than pneumonia (*p* = 0.201), when restricting the comparison to those received a primary diagnosis of COPD compared to those who received a primary diagnosis of pneumonia, a significant difference in the length of stay was observed (*p*<0.001)﻿, with those diagnosed with COPD having a length of stay of 3 days (IQR 1–7 days) compared to those with a primary diagnosis of pneumonia (5 days, IQR 2–9 days).

**Table 1:** Characteristics of patients with an eligible pneumonia diagnosis in primary care grouped according to whether patients were admitted to hospital within 7 days and whether patients received a pneumonia diagnosis in hospital

**Variable**	**Not admitted to hospital within 7 days (*N* = 5,466)** **N (%)**	**Admitted to hospital within 7 days with diagnosis other than pneumonia (*N* = 886)** **N (%)**	**Admitted to hospital within 7 days with pneumonia diagnosis** **(*N* = 1,208)** **N (%)**	***P***
Gender				
Male	2954 (54.0)	456 (51.5)	678 (56.1)	0.107
Female	2512 (46.0)	430 (48.5)	530 (43.9)	
Age (years)				
Mean (SD)	74.4 (10.7)	76.0 (10.2)	75.9 (10.2)	<0.001
IMD quintile				
1	795 (14.5)	134 (15.1)	166 (13.7)	0.071
2	888 (16.2)	142 (16.0)	219 (18.1)	
3	1026 (18.8)	180 (20.3)	266 (22.0)	
4	1184 (21.7)	178 (20.1)	257 (21.3)	
5	1567 (28.7)	252 (28.4)	300 (24.8)	
Missing IMD quintile	6 (0.1)	0 (0.0)	0 (0.0)	
Smoking status				
Current smoker	2026 (37.1)	291 (32.8)	404 (33.4)	< 0.001
Ex-smoker	2900 (53.1)	593 (66.9)	799 (66.1)	
No evidence of smoking history	540 (9.9)	2 (0.2)	5 (0.4)	
BMI category				
Underweight	428 (7.8)	68 (7.7)	96 (7.9)	0.996
Normal	1922 (35.2)	308 (34.8)	432 (35.8)	
Overweight	1646 (30.1)	269 (30.4)	366 (30.3)	
Obese	1394 (25.5)	230 (26.0)	301 (24.9)	
Missing	76 (1.4)	11 (1.2)	13 (1.1)	
Diastolic blood pressure (mmHg)				
Mean (SD)	72.5 (10.9)	72.0 (11.0)	71.4 (11.4)	0.004
Systolic blood pressure (mmHg)				
Mean (SD)	128.4 (17.6)	127.3 (20.0)	126.1 (18.6)	< 0.001
Hypertension				
Yes	2918 (53.4)	504 (56.9)	675 (55.9)	0.067
No	2548 (46.6)	382 (43.1)	533 (44.1)	
GOLD status				
Gold stage 1: > = 80%	967 (17.7)	147 (16.6)	198 (16.4)	0.861
Gold stage 2: 50–79%	2445 (44.7)	388 (43.8)	537 (44.5)	
Gold stage 3: 30–49%	1370 (25.1)	232 (26.2)	316 (26.2)	
Gold stage 4: < 30%	333 (6.1)	56 (6.3)	83 (6.9)	
Missing FEV1%-pred measurement	351 (6.4)	63 (7.1)	74 (6.1)	
Any malignancy, including leukemia and lymphoma (CCI)				
Yes	1465 (26.8)	276 (31.2)	362 (30.0)	0.005
No	4001 (73.2)	610 (68.8)	846 (70.0)	
Cerebrovascular disease (CCI)				
Yes	1014 (18.6)	199 (22.5)	230 (19.0)	0.023
No	4452 (81.4)	687 (77.5)	978 (81.0)	
Congestive heart failure (CCI)				
Yes	932 (17.1)	189 (21.3)	222 (18.4)	0.007
No	4534 (82.9)	697 (78.7)	986 (81.6)	
Dementia (CCI)				
Yes	489 (8.9)	97 (10.9)	108 (8.9)	0.152
No	4977 (91.1)	789 (89.1)	1100 (91.1)	
Diabetes without chronic complications (CCI)				
Yes	1172 (21.4)	188 (21.2)	251 (20.8)	0.876
No	4294 (78.6)	698 (78.8)	957 (79.2)	
Diabetes with chronic complications (CCI)				
Yes	692 (12.7)	124 (14.0)	163 (13.5)	0.453
No	4774 (87.3)	762 (86.0)	1045 (86.5)	
AIDS/HIV (CCI)				
Yes	30 (0.5)	1 (0.1)	1 (0.1)	0.025
No	5436 (99.5)	885 (99.9)	1207 (99.9)	
Hemiplegia or paraplegia (CCI)				
Yes	51 (0.9)	9 (1.0)	14 (1.2)	0.765
No	5415 (99.1)	877 (99.0)	1194 (98.8)	
Metastatic solid tumor (CCI)				
Yes	86 (1.6)	18 (2.0)	18 (1.5)	0.564
No	5380 (98.4)	868 (98.0)	1190 (98.5)	
Mild liver disease (CCI)				
Yes	134 (2.5)	19 (2.1)	24 (2.0)	0.576
No	5332 (97.5)	867 (97.9)	1184 (98.0)	
Moderate or severe liver disease (CCI)				
Yes	32 (0.6)	3 (0.3)	4 (0.3)	0.394
No	5434 (99.4)	883 (99.7)	1204 (99.7)	
Myocardial infarction (CCI)				
Yes	681 (12.5)	111 (12.5)	147 (12.2)	0.957
No	4785 (87.5)	775 (87.5)	1061 (87.8)	
Peptic ulcer disease (CCI)				
Yes	511 (9.3)	92 (10.4)	116 (9.6)	0.618
No	4955 (90.7)	794 (89.6)	1092 (90.4)	
Peripheral vascular disease (CCI)				
Yes	850 (15.6)	119 (13.4)	197 (16.3)	0.175
No	4616 (84.4)	767 (86.6)	1011 (83.7)	
Renal disease (CCI)				
Yes	1455 (26.6)	294 (33.2)	366 (30.3)	< 0.001
No	4011 (73.4)	592 (66.8)	842 (69.7)	
Rheumatologic disease (CCI)				
Yes	566 (10.4)	106 (12.0)	154 (12.7)	0.031
No	4900 (89.6)	780 (88.0)	1054 (87.3)	
Total number of comorbidities				
Mean (SD)	2.9 (1.5)	3.1 (1.5)	3.0 (1.5)	< 0.001
CCI score				
Mean (SD)	2.8 (1.8)	3.1 (1.8)	2.9 (1.8)	< 0.001
Asthma diagnosis				
Yes	1562 (28.6)	229 (25.8)	338 (28.0)	0.243
No	3904 (71.4)	657 (74.2)	870 (72.0)	
Anxiety				
Yes	1713 (31.3)	250 (28.2)	330 (27.3)	0.008
No	3753 (68.7)	636 (71.8)	878 (72.7)	
Depression				
Yes	1798 (32.9)	283 (31.9)	383 (31.7)	0.660
No	3668 (67.1)	603 (68.1)	825 (68.3)	
LAMA-LABA dual therapy prescribed in the 5 years preceding pneumonia diagnosis				
Yes	583 (10.7)	112 (12.6)	122 (10.1)	0.147
No	4883 (89.3)	774 (87.4)	1086 (89.9)	
ICS-LABA dual therapy prescribed in the 5 years preceding pneumonia diagnosis				
Yes	272 (5.0)	41 (4.6)	50 (4.1)	0.453
No	5194 (95.0)	845 (95.4)	1158 (95.9)	
LAMA-LABA-ICS triple therapy prescribed in the 5 years preceding pneumonia diagnosis				
Yes	638 (11.7)	95 (10.7)	140 (11.6)	0.713
No	4828 (88.3)	791 (89.3)	1068 (88.4)	
LAMA therapy prescribed in the 5 years preceding pneumonia diagnosis				
Yes	4118 (75.3)	693 (78.2)	936 (77.5)	0.076
No	1348 (24.7)	193 (21.8)	272 (22.5)	
LABA therapy prescribed in the 5 years preceding pneumonia diagnosis				
Yes	1273 (23.3)	221 (24.9)	272 (22.5)	0.420
No	4193 (76.7)	665 (75.1)	936 (77.5)	
ICS therapy prescribed in the 5 years preceding pneumonia diagnosis				
Yes	4433 (81.1)	703 (79.3)	999 (82.7)	0.150
No	1033 (18.9)	183 (20.7)	209 (17.3)	
Oral corticosteroids prescribed in the 5 years preceding pneumonia diagnosis				
Yes	4241 (77.6)	684 (77.2)	958 (79.3)	0.385
No	1225 (22.4)	202 (22.8)	250 (20.7)	
SABA prescribed in the 5 years preceding pneumonia diagnosis				
Yes	5091 (93.1)	823 (92.9)	1141 (94.5)	0.219
No	375 (6.9)	63 (7.1)	67 (5.5)	
SAMA prescribed in the 5 years preceding pneumonia diagnosis				
Yes	498 (9.1)	74 (8.4)	113 (9.4)	0.711
No	4968 (90.9)	812 (91.6)	1095 (90.6)	
Length of stay in hospital (days)				
Median (IQR)	-	5.0 (1.0 to 11.0)	5.0 (2.0 to 9.0)	0.204

**Table 2:** Assessing the positive predictive value of pneumonia coding in primary care for predicting pneumonia diagnosis in hospital for those admitted to hospital within 7 days of diagnosis. Low numbers of events have been censored

**Pneumonia identification algorithm in primary care**	**Eligible events identified** **N**	**Admitted to hospital within 7 days** **N (%)**	**Primary diagnosis of pneumonia in hospital (ICD10 codes J12-J18)** **N (%)**	**Positive predictive value** **% (95% CI)**^**a**^
Pneumonia code only	7,560	2,094 (27.7%)	1,208 (57.7%)	57.7 (55.6–59.8)
Pneumonia code and symptoms of pneumonia (include two of the following symptoms, new cough, sputum, lethargy, fever, tachycardia, breathlessness)	242	84 (34.7%)	52 (61.9%)	61.9 (51.2–71.6)
Pneumonia code and referral for chest X-ray	1,027	344 (33.5%)	207 (60.2%)	60.2 (54.9–65.2)
Pneumonia code and evidence of sputum or blood culture sent	229	78 (34.1%)	49 (62.8%)	62.8 (51.7–72.7)
Pneumonia code and evidence of sputum or blood culture positive result	13	< 5 (30.8%)	< 5 (100.0%)	100.0 (51.0–100.0)
Pneumonia code and symptoms of pneumonia and referral for chest X-ray	68	18 (26.5%)	8 (44.4%)	44.4 (24.6–66.3)
Pneumonia code and antibiotics use (antibiotic prescription of 5–14 days)	1,638	303 (18.5%)	144 (47.5%)	47.5 (42.0–53.1)
Pneumonia code and antibiotics use (antibiotic prescription of any duration)	2,443	529 (21.7%)	274 (51.8%)	51.8 (47.5–56.0)
Pneumonia code, symptoms, and antibiotics	98	28 (28.6%)	15 (53.6%)	53.6 (35.8–70.5)
Pneumonia code, referral for chest X-ray, and antibiotics	237	49 (20.7%)	25 (51.0%)	51.0 (37.5–64.4)
Pneumonia code, antibiotics and evidence of sputum or blood culture sent	99	26 (26.3%)	14 (53.8%)	53.8 (35.5–71.2)
Pneumonia code, antibiotics and evidence of sputum or blood culture positive result	< 5	< 5 (50.0%)	< 5 (100.0%)	100.0 (20.7–100.0)
Pneumonia code, referral for chest X-ray, and evidence of sputum or blood culture sent	45	11 (24.4%)	6 (54.5%)	54.5 (28.0–78.7)
Pneumonia code, referral for chest X-ray, and evidence of sputum or blood culture positive result	7	< 5 (14.3%)	< 5 (100.0%)	100.0 (20.7–100.0)
Pneumonia code, symptoms of pneumonia and evidence of sputum or blood culture sent	184	68 (37.0%)	44 (64.7%)	64.7 (52.8–75.0)
Pneumonia code, symptoms of pneumonia and evidence of sputum or blood culture positive result	7	< 5 (42.9%)	< 5 (100.0%)	100.0 (43.9–100.0)
Pneumonia code, referral for chest X-ray, symptoms of pneumonia and antibiotics	16	< 5 (18.8%)	0 (0.0%)	0.0 (0.0–56.1)
Pneumonia code, referral for chest X-ray, symptoms of pneumonia and antibiotics and evidence of sputum or blood culture sent	11	< 5 (9.1%)	0 (0.0%)	0.0 (0.0–79.3)
Pneumonia code, referral for chest X-ray, symptoms of pneumonia and antibiotics and evidence of sputum or blood culture positive result	0	0	0	-

^a^The PPV corresponds to the percentage of patients that receive a primary diagnosis of pneumonia in hospital (preceding column), but is presented with confidence intervals here for clarity

A sensitivity analysis which used pneumonia diagnosis in any position in the final episode as the gold standard increased the PPV of pneumonia code in primary care to 67.5% (95% CI 65.5–69.5). A sensitivity analysis which restricted PPV calculation to just those that included same-day admissions increased the PPV to 65.8% (95% CI 63.3–68.2). When restricted to same-day admissions with pneumonia diagnosis in any position as the gold standard, the PPV was increased to 75.9% (95% CI 73.6–78.0). Full results for all algorithms can be found in the Supplemental materials in Table 1, 2 and 3.

The characteristics of patients who had an eligible pneumonia event in secondary care are displayed in Table 3. Those who had a recording of pneumonia in primary care within 42 days tended to be younger, more overweight, and at an earlier GOLD stage, but with a similar level of comorbidity. Only 11,445/33,603 patients had a recording of pneumonia in primary care in the 42 days following hospitalisation. This represents a sensitivity of 34.1% (95% CI 33.6%-34.6%). After restricting to pneumonia code together with a generic or respiratory hospitalisation code on the same day, the sensitivity was reduced to 20.3% (95% CI 19.8%–20.7%). The breakdown of the most common pneumonia codes used to record secondary care pneumonia can be found in Supplementary Fig. 2.

**Table 3:** Characteristics of patients with an eligible pneumonia diagnosis in secondary care

**Variable**	**Pneumonia not recorded in primary care within 42 days (*N* = 22,158) N (%)**	**Pneumonia recorded in primary care within 42 days (*N* = 11,445) N (%)**	***P***
Gender			
Male	11,963 (54.0)	6189 (54.1)	0.889
Female	10,195 (46.0)	5256 (45.9)	
Age (years)			
Mean (SD)	76.1 (10.2)	75.4 (10.2)	< 0.001
IMD quintile			
1	876 (4.0)	1605 (14.0)	< 0.001
2	1044 (4.7)	1838 (16.1)	
3	1231 (5.6)	2124 (18.6)	
4	1476 (6.7)	2539 (22.2)	
5	1913 (8.6)	3331 (29.1)	
Missing IMD quintile	15,618 (70.5)	8 (0.1)	
Smoking status			
Current smoker	8248 (37.2)	4045 (35.3)	0.003
Ex-smoker	13,847 (62.5)	7371 (64.4)	
No evidence of smoking history	63 (0.3)	29 (0.3)	
BMI category			
Underweight	2188 (9.9)	891 (7.8)	< 0.001
Normal	8117 (36.6)	4039 (35.3)	
Overweight	6124 (27.6)	3362 (29.4)	
Obese	5401 (24.4)	3008 (26.3)	
Missing	328 (1.5)	145 (1.3)	
Diastolic blood pressure (mmHg)			
Mean (SD)	72.2 (11.0)	72.1 (11.0)	0.370
Systolic blood pressure (mmHg)			
Mean (SD)	127.6 (18.3)	127.8 (18.4)	0.526
Hypertension			
Yes	12,286 (55.4)	6262 (54.7)	0.204
No	9872 (44.6)	5183 (45.3)	
GOLD status			
Gold stage 1: > = 80%	2948 (13.3)	1848 (16.1)	< 0.001
Gold stage 2: 50–79%	9032 (40.8)	4971 (43.4)	
Gold stage 3: 30–49%	6572 (29.7)	3085 (27.0)	
Gold stage 4: < 30%	2168 (9.8)	834 (7.3)	
Missing FEV1%-pred measurement	1438 (6.5)	707 (6.2)	
Any malignancy, including leukemia and lymphoma (CCI)			
Yes	6330 (28.6)	3282 (28.7)	0.844
No	15,828 (71.4)	8163 (71.3)	
Cerebrovascular disease (CCI)			
Yes	4350 (19.6)	2231 (19.5)	0.773
No	17,808 (80.4)	9214 (80.5)	
Congestive heart failure (CCI)			
Yes	4087 (18.4)	2073 (18.1)	0.465
No	18,071 (81.6)	9372 (81.9)	
Dementia (CCI)			
Yes	2136 (9.6)	1024 (8.9)	0.041
No	20,022 (90.4)	10,421 (91.1)	
Diabetes without chronic complications (CCI)			
Yes	4666 (21.1)	2342 (20.5)	0.209
No	17,492 (78.9)	9103 (79.5)	
Diabetes with chronic complications (CCI)			
Yes	2838 (12.8)	1524 (13.3)	0.195
No	19,320 (87.2)	9921 (86.7)	
AIDS/HIV (CCI)			
Yes	43 (0.2)	24 (0.2)	0.861
No	22,115 (99.8)	11,421 (99.8)	
Hemiplegia or paraplegia (CCI)			
Yes	191 (0.9)	101 (0.9)	0.897
No	21,967 (99.1)	11,344 (99.1)	
Metastatic solid tumor (CCI)			
Yes	390 (1.8)	215 (1.9)	0.465
No	21,768 (98.2)	11,230 (98.1)	
Mild liver disease (CCI)			
Yes	410 (1.9)	223 (1.9)	0.559
No	21,748 (98.1)	11,222 (98.1)	
Moderate or severe liver disease (CCI)			
Yes	85 (0.4)	62 (0.5)	0.046
No	22,073 (99.6)	11,383 (99.5)	
Myocardial infarction (CCI)			
Yes	2809 (12.7)	1579 (13.8)	0.004
No	19,349 (87.3)	9866 (86.2)	
Peptic ulcer disease (CCI)			
Yes	2055 (9.3)	1152 (10.1)	0.020
No	20,103 (90.7)	10,293 (89.9)	
Peripheral vascular disease (CCI)			
Yes	3484 (15.7)	1878 (16.4)	0.107
No	18,674 (84.3)	9567 (83.6)	
Renal disease (CCI)			
Yes	6403 (28.9)	3210 (28.0)	0.105
No	15,755 (71.1)	8235 (72.0)	
Rheumatologic disease (CCI)			
Yes	2100 (9.5)	1240 (10.8)	< 0.001
No	20,058 (90.5)	10,205 (89.2)	
Total number of comorbidities			
Mean (SD)	2.9 (1.5)	2.9 (1.5)	0.164
CCI score			
Mean (SD)	2.8 (1.8)	2.8 (1.8)	0.689
Asthma diagnosis			
Yes	5372 (24.2)	2982 (26.1)	< 0.001
No	16,786 (75.8)	8463 (73.9)	
Anxiety			
Yes	6080 (27.4)	3207 (28.0)	0.264
No	16,078 (72.6)	8238 (72.0)	
Depression			
Yes	6756 (30.5)	3579 (31.3)	0.145
No	15,402 (69.5)	7866 (68.7)	
LAMA-LABA dual therapy prescribed in the 5 years preceding pneumonia diagnosis			
0	19,962 (90.1)	10,227 (89.4)	0.037
1	2196 (9.9)	1218 (10.6)	
ICS-LABA dual therapy prescribed in the 5 years preceding pneumonia diagnosis			
Yes	856 (3.9)	509 (4.4)	0.011
No	21,302 (96.1)	10,936 (95.6)	
LAMA-LABA-ICS triple therapy prescribed in the 5 years preceding pneumonia diagnosis			
Yes	2267 (10.2)	1223 (10.7)	0.202
No	19,891 (89.8)	10,222 (89.3)	
LAMA therapy prescribed in the 5 years preceding pneumonia diagnosis			
Yes	17,320 (78.2)	8739 (76.4)	< 0.001
No	4838 (21.8)	2706 (23.6)	
LABA therapy prescribed in the 5 years preceding pneumonia diagnosis			
Yes	4634 (20.9)	2552 (22.3)	0.004
No	17,524 (79.1)	8893 (77.7)	
ICS therapy prescribed in the 5 years preceding pneumonia diagnosis			
Yes	18,292 (82.6)	9279 (81.1)	0.001
No	3866 (17.4)	2166 (18.9)	
Oral corticosteroids prescribed in the 5 years preceding pneumonia diagnosis			
Yes	17,503 (79.0)	9009 (78.7)	0.566
No	4655 (21.0)	2436 (21.3)	
SABA prescribed in the 5 years preceding pneumonia diagnosis			
Yes	20,771 (93.7)	10,735 (93.8)	0.859
No	1387 (6.3)	710 (6.2)	
SAMA prescribed in the 5 years preceding pneumonia diagnosis			
Yes	2236 (10.1)	1062 (9.3)	0.019
No	19,922 (89.9)	10,383 (90.7)	
Length of stay in hospital (days)			
Median (IQR)	6 (3 to 12)	5 (3 to 10)	0.001

## Discussion

Pneumonia coding in general practice for more serious events that result in admission to hospital have a reasonable PPV of 58% but misdiagnosis does occur, with 14% of patients with a diagnosis of pneumonia in primary care admitted to hospital with a COPD respiratory code and 5% admitted with a non-COPD respiratory code. PPV increased to 68% when allowing pneumonia diagnosis in any position. Including additional factors such as antibiotic prescriptions changed the PPV but markedly reduced the number of events identified and so is not recommended. When assessing the percentage of hospitalisations that are recorded in primary care, we found that only 34% were recorded in primary care within 42 days using pneumonia code only, decreasing to 20.3% when restricting to pneumonia code with associated hospitalisation code. Given that all hospitalisations should be recorded in primary care, this is a concerning finding. This study has found that pneumonia codes in primary care are not suitable for assessing pneumonia events in COPD patients, due to the common overlap between LRTI and pneumonia in this population and the fact that many hospitalisations are missed. Moreover, 30–40% of GP-coded pneumonia that results in a hospital admission is not diagnosed as pneumonia in hospital, and those that were given a primary diagnosis of COPD in hospital had a significantly shorter length of stay than those with a diagnosis of pneumonia. For GP-recorded pneumonia that does not result in hospital admission, this study was not able to assess the quality of recording but our results are suggestive of this being poorly recorded if severe (and hence more easily diagnosed) pneumonia is only confirmed in hospital 60–70% of the time. For this reason, we advise using pneumonia hospitalisations only for all studies with pneumonia as an outcome in a COPD patient population.

We have shown that pneumonia events diagnosed in primary care in COPD patients are often not diagnosed as pneumonia in hospital, and that attempts to increase accuracy of pneumonia identification in primary care by including other variables such as prescription of antibiotics and referral for chest X-ray in primary care is not recommended as it will result in significant underestimates of prevalence. This is particularly applicable when assessing the risk of pneumonia when ICS is prescribed to COPD patients. Recent NICE guidance [10] assessing the effectiveness of LABA-LAMA-ICS triple therapy in treating COPD versus LABA-LAMA and LABA-ICS dual therapy included pneumonia as a secondary outcome, due to the association between ICS and pneumonia risk in COPD patients [16]. Of the three studies which included the comparison between triple therapy and LABA-LAMA dual therapy [17–19], two required pneumonia events to be confirmed by chest radiograph as part of the case definition to minimise misdiagnosis [17, 19]. One study, which made up 11.8% of the meta-analysis weighting, required investigators to “undertake, whenever possible, further investigations based on their clinical experience and judgement” when defining pneumonia but did not explicitly require radiographic confirmation [18]. It is possible that this study may have included misclassified GP-diagnosed pneumonia events without associated chest X-rays, however the low weighting given to this study means that the overall association between ICS and pneumonia in the meta-analysis would not be altered even if misclassification was present. The increase in pneumonia risk for triple therapy versus LABA-LAMA dual therapy corresponds to that seen for ICS only or ICS-LABA dual therapy verses LABA single therapy or placebo [20].

In observational studies, and particularly those using routinely collected electronic healthcare data where it is not possible to collect additional data such as chest X-rays, researchers must be especially cautious when defining outcomes. Our study helps to reiterate the importance of the vigorous case definition generally used by RCTs and we would recommend researchers assessing pneumonia risk in COPD patients in EHR use hospitalised pneumonia only. Furthermore, due to poor recording of hospitalised pneumonia in general practice, hospitalised pneumonia should be identified using hospital data rather than indirectly using GP-collected data. This is the approach taken by many observational studies (e.g. [21, 22]), which often include hospitalised pneumonia alone or GP-recorded pneumonia in tandem with hospitalised pneumonia [23–26]. Studies carried out in primary care databases such as CPRD require additional linkage with hospital data to do this, and not all studies follow this recommendation, for example [27, 28]. This can cause issues if pneumonia is differentially diagnosed over LRTI by GPs aware that ICS use is associated with an increased risk of pneumonia.

Understanding the quality of pneumonia coding in primary care is challenging and studies have approached this in a variety of ways. Merepol and Metlay [29] assessed the PPV of GP-assessed pneumonia together with codes indicating hospitalisation in The Health Improvement Network (THIN) database, using pneumonia assessed using all hospitalisation documentation as the gold standard. They found that GP-assessed pneumonia codes together with codes indicating hospitalisation had a PPV of 86% (51 of 59; 95%CI = 75%–94%) for hospitalisation with pneumonia within 30 days of GP code. This is slightly different to our method, in that it measures the quality of GP recording of hospitalised pneumonia indicating a true hospitalisation rather than the sensitivity of GP-recorded hospitalised pneumonia identifying true hospitalisation events. A study that more closely reflects ours aims [30] was carried out in the US, with the researchers attempting to assess how well pneumonia codes used for claims data reflected true pneumonia diagnosis across the healthcare system using patient medical records. They found a PPV that was higher than ours in outpatient settings, at 73.4% (149 of 203; 95% CI 66.8%–79.3%), however they note that chest X-ray was only present in 61.1% of cases so it is difficult to ascertain the accuracy of the diagnosis even with access to medical notes.

Interestingly, in our study we did not find that adding in additional clinical or treatment codes noticeably improved the PPV of a pneumonia diagnosis, despite evidence that these factors are useful in predicting pneumonia [31]. This may simply be because symptoms were under-recorded in our study and we did not have the power to detect a true difference in PPV. Under-recording of symptoms tends be common in EHR data, and is one of the limitations of using routinely collected healthcare data rather than data collected specifically for the purposes of research. We would posit that even if the PPV was improved, the associated drop in sensitivity would negate any benefits of the addition of symptoms. For antibiotic use, the PPV appeared to drop – this corroborates with the results found by Millet et al. [32]that receipt of antibiotics prescription in the previous 8–28 days was associated with a drop in the likelihood of hospitalisation. The lowered PPV could be due to increased clearance of infection in those prescribed antibiotics, or could reflect that the severity of suspected pneumonia was so great that the patient was advised to attend hospital directly without prescription.

Primary diagnosis of pneumonia in hospital was used as the gold standard in our study due to the availability of chest X-rays to make a definitive diagnosis. However, COPD patients present a particular diagnostic challenge due to the similarities in symptoms of AECOPD and pneumonia. A study comparing the discharge diagnosis with pneumonia defined as the presence of radiographic consolidation found that only 16% of COPD patients admitted to hospital with a respiratory illness had a discharge diagnosis of pneumonia despite a presence of radiographic consolidation in 25% of patients [33]. The authors argue that this “confusion stems from two different diagnostic approaches that can be taken in these patients; either to consider pneumonia as the primary diagnosis and COPD as a comorbidity or to consider COPD exacerbation as the primary diagnosis and pneumonia as a cause of the exacerbation”. When the definition of pneumonia was relaxed to include pneumonia coded in any position, we found that our PPV increased from 58 to 68%. Discrepancies in pneumonia diagnoses given to COPD patients may go some way towards explaining the low rates of recording of hospitalised pneumonia in primary care following hospitalisation that we found in our study, with pneumonia discharges in hospital possibly being recorded in primary case as AECOPD rather than pneumonia, although it has been found that AECOPD hospitalisations are also under-recorded in primary care [34].

We have validated pneumonia codes in patients in primary care who were later admitted to hospital, using the hospital admission as the gold standard due to the clinical diagnostic equipment available in hospital. This allows us a glimpse of the accuracy of coding in the field. We were able to assess a variety of coding algorithms to maximise the potential of the data available in the dataset. Whilst some algorithms such as symptoms codes and x-ray referral codes did increase the PPV of identifying pneumonia, albeit with greater uncertainty around the PPV point estimates, the total number of events identified sharply decreased, likely negating the usefulness of these more precise codes. The large number of patients in CPRD allowed us to maximise the accuracy of our analysis by giving us scope to restrict the admissions we study to just those that were observed and entered on the same day to assess the reporting of pneumonia diagnoses in primary care that then occur in secondary care, rather than vice versa.

To identify pneumonia, we used the last episode of the patient’s admission, in contrast to some other studies in this area which use the first episode [32]. This was used to minimise the abundance of non-specific respiratory symptom codes that can be entered for the first episode before a more specific diagnosis is reached. The drawback of using the last episode rather than the first is that we could identify hospital-acquired pneumonia rather than community-acquired pneumonia. We believe that we have mitigated this risk by the precautions we took to identify patients with pneumonia in primary care who are then prospectively admitted to secondary care, making it unlikely that a patient with a diagnosis of pneumonia in primary care would then be admitted to hospital with a different ailment and acquire pneumonia in hospital. When assessing the recording of hospitalised pneumonia in primary care, it was not necessary to restrict this to community-acquired pneumonia only. To assess recording in GP record within 42 days, we used the patients’ admission date rather than the discharge date, to ensure that hospitalised pneumonia dates relayed to the GP practice before discharge were not missed. If the pneumonia admission is relayed to the GP practice after discharge, this could result in patients with longer stays being less likely to receive a pneumonia record in primary care within 42 days. The median length of stay was similar in both groups (5 days in those with a recording in primary care and 6 days in those without a recording in primary care), so we do not expect that length of stay in hospital had a large effect on our analysis.

We have made every effort in our study to obtain as accurate diagnosis of pneumonia as possible, by using pneumonia diagnosed in hospital as the gold standard due to the availability of chest X-rays in hospital to obtain a definitive diagnosis. Whilst every care has been taken to only include pneumonia events in primary care that occurred before hospitalisation, by restricting to just those events which occur and are entered on the same day, it is possible that we may have identified some hospitalised events retrospectively recorded in primary care if a patient was admitted and discharged from hospital on the same day or if a hospital informed the patient’s GP in about the patient’s admission to hospital on the same day that it occurred. Whilst we consider both of these events to be unlikely, if this did occur then it would likely result in a PPV that is higher than the true value as recording of pneumonia post-hospitalisation is expected to be more accurate than pre-hospitalisation.

One drawback of our method is that we can only identify the PPV of primary care pneumonia diagnosis in those who are then admitted to hospital. In addition to documented confusion as to the coding of pneumonia in COPD patients [33], the use of hospitalised pneumonia as a gold standard results in only patients with illness that is severe enough to require hospitalisation being included. This means that our PPV is likely to be a maximum value if we consider than severe pneumonia is easier to diagnosis in primary care than severe pneumonia. Furthermore, it is not possible to calculate the sensitivity or negative predictive value of pneumonia coding in primary care because not all patients hospitalised with pneumonia will have attended primary care first (and so false negatives (those who are misdiagnosed as not having pneumonia in primary care) are not available). Lastly, it is possible that after pneumonia diagnosis in primary care, patients are in fact admitted to hospital in the next seven days for a separate reason. This may explain the increase in PPV in the sensitivity analysis in which we restricted to just events that occurred in primary care and secondary care on the same day.

Whilst we considered including AECOPD or LRTI codes in primary care as ‘negative for pneumonia’ to obtain an estimate of sensitivity, there are a number of drawbacks with this approach, as 1) it is possible for AECOPD to progress into pneumonia; 2) when identifying AECOPD and pneumonia in any position in hospital, the two diagnoses will no longer be mutually exclusive; and 3) it is unclear how this approach would work when using the different coding algorithms for pneumonia. A future study in which patients diagnosed with pneumonia in primary care receive a chest X-ray to confirm the diagnosis would remove some of these limitations, although this may not be ethically viable as use of chest X-rays in primary care to obtain a definitive diagnosis for suspected pneumonia is recommended against in primary care in the NICE guidelines [7].

## Conclusion

Whilst the addition of extra coding information such as chest X-ray referral and pneumonia symptoms along with a pneumonia code in primary care may increase the PPV, this is largely offset by the reduction in identified cases. Pneumonia code alone has a PPV of 58% when compared with pneumonia diagnosis in hospital, increasing to 75% when restricting to pneumonia diagnosed by the GP on the same day as hospital admission and classing hospital admissions with pneumonia code in any position as pneumonia. We found that only 34% of hospitalised pneumonia was recorded in primary care within 42 days. This leads us to recommend use of pneumonia diagnosed in hospital as the gold standard for identifying pneumonia events rather than those that are diagnosed in primary care alone.

### Supplementary Information

**Additional file 1.** This csv includes codes used for defining pneumonia and LRTI.

**Additional file 2: Supplemental Table 1.** Table providing data on the PPV of algorithms assessing pneumonia diagnosis in primary care when the gold standard definition for pneumonia in secondary care is extended to include all diagnosis positions.

**Additional file 3: Supplemental Table 2.** Table providing data on the PPV of algorithms assessing pneumonia diagnosis in primary care when only looking at same-day hospitalisations.

**Additional file 4: Supplemental Table 3.** Table providing data on the PPV of algorithms assessing pneumonia diagnosis in primary care when the gold standard definition for pneumonia in secondary care is extended to include all diagnosis positions, looking only at same-day hospitalisations.

**Additional file 5: Supplementary Figures.** Figures showing the breakdown of the GP-coded pneumonia terms used in the analysis.

## Data Availability

CPRD has NHS Health Research Authority (HRA) Research Ethics Committee (REC) approval to allow the collection and release of anonymised primary care data for observational research [NHS HRA REC reference number: 05/MRE04/87]. Each year CPRD obtains Section 251 regulatory support through the HRA Confidentiality Advisory Group (CAG), to enable patient identifiers, without accompanying clinical data, to flow from CPRD contributing GP practices in England to NHS Digital, for the purposes of data linkage [CAG reference number: 21/CAG/0008]. The protocol for this research was approved by CPRD’s Research Data Governance (RDG) Process (protocol number: #21_000468) and the approved protocol is available upon request. Linked pseudonymised data was provided for this study by CPRD. Data is linked by NHS Digital, the statutory trusted third party for linking data, using identifiable data held only by NHS Digital. Select general practices consent to this process at a practice level with individual patients having the right to opt-out. This study is based in part on data from the Clinical Practice Research Datalink obtained under licence from the UK Medicines and Healthcare products Regulatory Agency. The data is provided by patients and collected by the NHS as part of their care and support. Hospital Episode Statistics (HES) was the provider of HES-Admitted Patient Care databases contained within the CPRD Data and maintain a Copyright © 2024. Linked data were re-used with the permission of The Health & Social Care Information Centre, all rights reserved. The interpretation and conclusions contained in this study are those of the author/s alone. Data are available on request from the CPRD. Their provision requires the purchase of a license, and this license does not permit the authors to make them publicly available to all. This work used data from the version collected in May 2021 and have clearly specified the data selected within each Methods section. To allow identical data to be obtained by others, via the purchase of a license, all analysis scripts and codelists are available at https://github.com/NHLI-Respiratory-Epi/Pneumonia-Accuracy-EHR. Licenses are available from the CPRD (http://www.cprd.com): The Clinical Practice Research Datalink Group, The Medicines and Healthcare products Regulatory Agency, 10 South Colonnade, Canary Wharf, London E14 4PU.
