# Supplementary material for: Accuracy of the recording of pneumonia events in English electronic healthcare record data in patients with chronic obstructive pulmonary disease
Source: Pneumonia (Nathan). 2024 May 5;16:8. doi: 10.1186/s41479-024-00130-2 (PMC11070075; doi:10.1186/s41479-024-00130-2)
Supplement: Supplementary file 4 — Additional file 4: Supplemental Table 3. Table providing data on the PPV of algorithms assessing pneumonia diagnosis in primary care when the gold standard definition for pneumonia in secondary care is extended to include all diagnosis positions, looking only at same-day hospitalisations. [file 41479_2024_130_MOESM4_ESM.docx]

**Supplemental Table 3** Assessing the positive predictive value of pneumonia coding in primary care for predicting pneumonia diagnosis in hospital in any position for those admitted to hospital on the same day as diagnosis. Low numbers of events have been censored.

| **Pneumonia identification algorithm in primary care** | **Eligible events identified**  **N** | **Admitted to hospital on the same day**  **N (%)** | **Diagnosis of pneumonia in hospital in any position (ICD10 codes J12-J18)**  **N (%)** | **Positive predictive value**  **% (95% CI)*** |
| --- | --- | --- | --- | --- |
| Pneumonia code only | 7560 | 1452 (19.2%) | 1102 (75.9%) | 75.9 (73.6 - 78.0) |
| Pneumonia code and symptoms of pneumonia (include two of the following symptoms, new cough, sputum, lethargy, fever, tachycardia, breathlessness) | 242 | 52 (21.5%) | 40 (76.9%) | 76.9 (63.9 - 86.3) |
| Pneumonia code and referral for chest X-ray | 1027 | 250 (24.3%) | 191 (76.4%) | 76.4 (70.8 - 81.2) |
| Pneumonia code and evidence of sputum or blood culture sent | 229 | 51 (22.3%) | 39 (76.5%) | 76.5 (63.2 - 86.0) |
| Pneumonia code and evidence of sputum or blood culture positive result | 13 | <5 (23.1%) | <5 (100.0%) | 100.0 (43.9 - 100.0) |
| Pneumonia code and symptoms of pneumonia and referral for chest X-ray | 68 | 10 (14.7%) | 6 (60.0%) | 60.0 (31.3 - 83.2) |
| Pneumonia code and antibiotics use (antibiotic prescription of 5-14 days) | 1638 | 138 (8.4%) | 84 (60.9%) | 60.9 (52.5 - 68.6) |
| Pneumonia code and antibiotics use (antibiotic prescription of any duration) | 2443 | 295 (12.1%) | 198 (67.1%) | 67.1 (61.6 - 72.2) |
| Pneumonia code, symptoms, and antibiotics | 98 | 16 (16.3%) | 12 (75.0%) | 75.0 (50.5 - 89.8) |
| Pneumonia code, referral for chest X-ray, and antibiotics | 237 | 28 (11.8%) | 14 (50.0%) | 50.0 (32.6 - 67.4) |
| Pneumonia code, antibiotics and evidence of sputum or blood culture sent | 99 | 16 (16.2%) | 12 (75.0%) | 75.0 (50.5 - 89.8) |
| Pneumonia code, antibiotics and evidence of sputum or blood culture positive result | <5 | <5 (50.0%) | <5 (100.0%) | 100.0 (20.7 - 100.0) |
| Pneumonia code, referral for chest X-ray, and evidence of sputum or blood culture sent | 45 | 7 (15.6%) | <5 (57.1%) | 57.1 (25.0 - 84.2) |
| Pneumonia code, referral for chest X-ray, and evidence of sputum or blood culture positive result | 7 | 0 (0.0%) | 0 | - |
| Pneumonia code, symptoms of pneumonia and evidence of sputum or blood culture sent | 184 | 43 (23.4%) | 33 (76.7%) | 76.7 (62.3 - 86.8) |
| Pneumonia code, symptoms of pneumonia and evidence of sputum or blood culture positive result | 7 | <5 (28.6%) | <5 (100.0%) | 100.0 (34.2 - 100.0) |
| Pneumonia code, referral for chest X-ray, symptoms of pneumonia and antibiotics | 16 | <5 (6.2%) | 0 (0.0%) | 0.0 (0.0 - 79.3) |
| Pneumonia code, referral for chest X-ray, symptoms of pneumonia and antibiotics and evidence of sputum or blood culture sent | 11 | <5 (9.1%) | 0 (0.0%) | 0.0 (0.0 - 79.3) |
| Pneumonia code, referral for chest X-ray, symptoms of pneumonia and antibiotics and evidence of sputum or blood culture positive result | 0 | 0 | 0 | - |

*The PPV corresponds to the percentage of patients that receive a primary diagnosis of pneumonia in hospital (preceding column), but is presented with confidence intervals here for clarity
