# Supplementary material for: Accuracy of the recording of pneumonia events in English electronic healthcare record data in patients with chronic obstructive pulmonary disease
Source: Pneumonia (Nathan). 2024 May 5;16:8. doi: 10.1186/s41479-024-00130-2 (PMC11070075; doi:10.1186/s41479-024-00130-2)

**Supplementary figure 1.** Frequency of pneumonia terms used in primary care that did or did not result in a pneumonia diagnosis in secondary care.

**
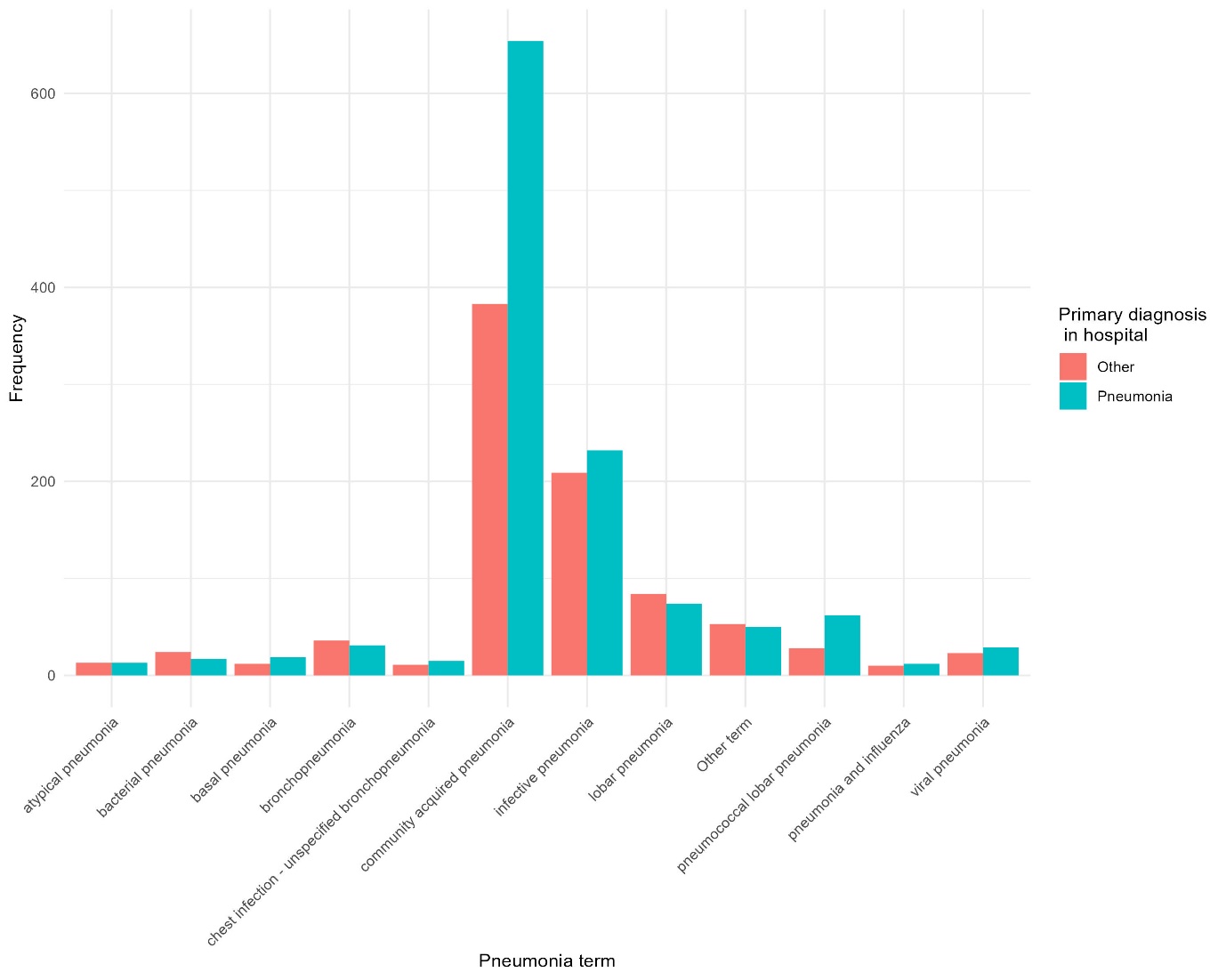
**

**Supplementary figure 2.** Frequency of pneumonia terms recorded in primary care after pneumonia hospitalisation.


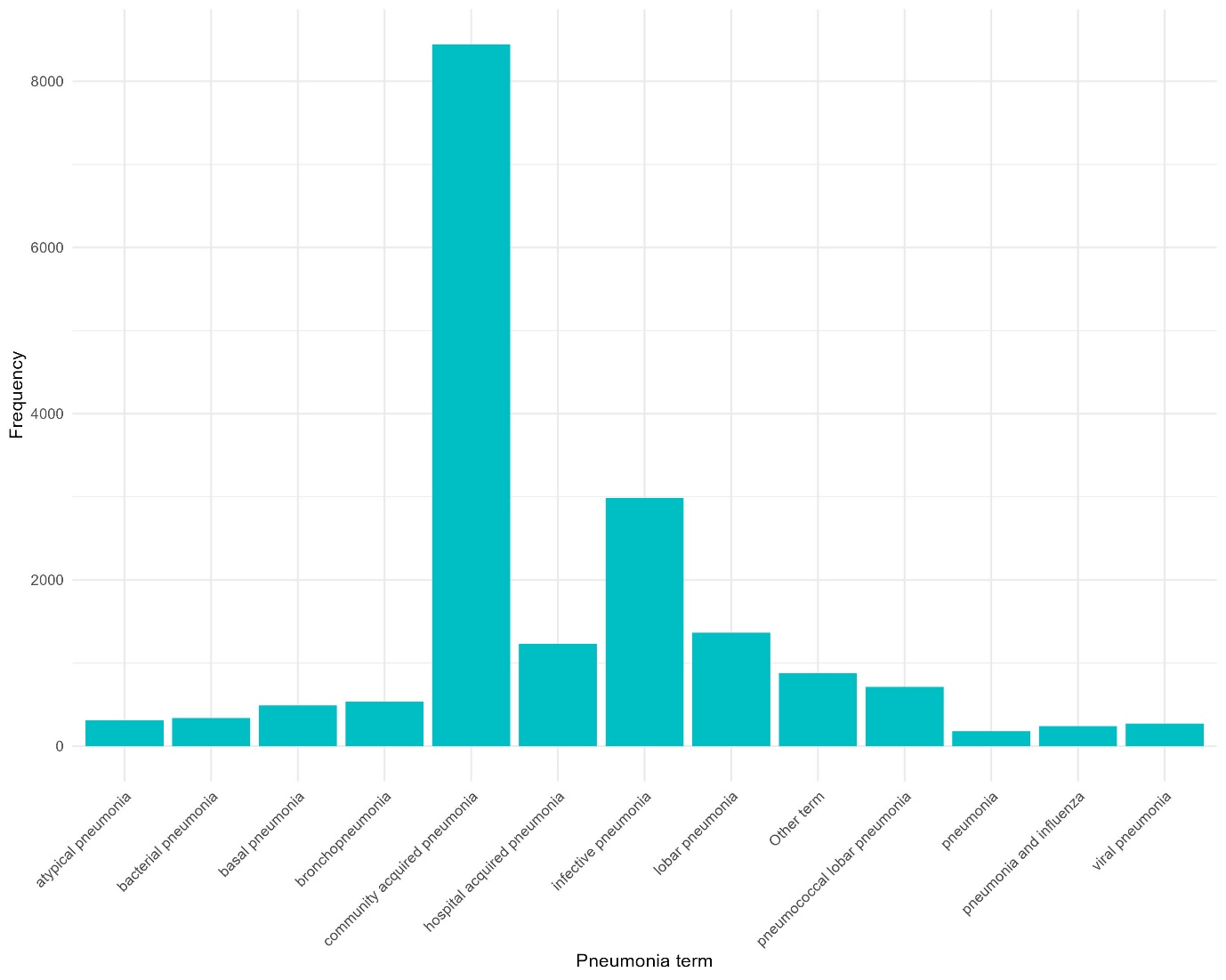

Supplement: Supplementary file 5 — Additional file 5: Supplementary Figures. Figures showing the breakdown of the GP-coded pneumonia terms used in the analysis. [file 41479_2024_130_MOESM5_ESM.docx]
